# Cellular complexity of the peripheral nervous system: Insights from single-cell resolution

**DOI:** 10.3389/fnins.2023.1098612

**Published:** 2023-03-14

**Authors:** Lili Zhao, Weixiao Huang, Sheng Yi

**Affiliations:** ^1^Key Laboratory of Neuroregeneration of Jiangsu and Ministry of Education, Co-Innovation Center of Neuroregeneration, Nantong University, Nantong, Jiangsu, China; ^2^NMPA Key Laboratory for Research and Evaluation of Tissue Engineering Technology Products, Nantong University, Nantong, Jiangsu, China; ^3^School of Medicine and Life Sciences, Nanjing University of Chinese Medicine, Nanjing, China

**Keywords:** heterogeneity, peripheral nervous system, regeneration, single-cell sequencing (scRNA-seq), nerve injury and regeneration

## Abstract

Single-cell RNA sequencing allows the division of cell populations, offers precise transcriptional profiling of individual cells, and fundamentally advances the comprehension of cellular diversity. In the peripheral nervous system (PNS), the application of single-cell RNA sequencing identifies multiple types of cells, including neurons, glial cells, ependymal cells, immune cells, and vascular cells. Sub-types of neurons and glial cells have further been recognized in nerve tissues, especially tissues in different physiological and pathological states. In the current article, we compile the heterogeneities of cells that have been reported in the PNS and describe cellular variability during development and regeneration. The discovery of the architecture of peripheral nerves benefits the understanding of the cellular complexity of the PNS and provides a considerable cellular basis for future genetic manipulation.

## 1. Introduction

The nervous system in mammals can be divided into the central nervous system (CNS) and the peripheral nervous system (PNS). The PNS, spreading over the body, transmits environmental information to the CNS and connects the CNS to target tissues and organs. Classically, the PNS is sub-classified into the somatic and autonomic nervous systems (Vermeiren et al., 2020). The somatic nervous system consists of motor and sensory neurons and is associated with conscious activities, such as the movements of extremities. The autonomic nervous system can be functionally distinguished into the sympathetic, parasympathetic, and enteric nervous systems. These classifications rely on the locations of neuronal somas and axons. Are the states and/or preferences of neurons in somatic and autonomic nervous systems similar, or do they have great differences? Not until the advent of single-cell sequencing in recent years has a definitive answer been provided.

The understanding of specific transcriptional characteristics has largely expanded since 2009 when Tang et al. (2009) developed a single-cell digital gene expression profiling assay. A large number of single-cell sequencing technologies have been developed and utilized since then, such as single-cell tagged reverse transcription sequencing (STRT-seq) (Islam et al., 2011) in 2011, a switching mechanism at the 5′ end of the RNA transcript (Smart-seq) (Ramskold et al., 2012) and cell expression by linear amplification and sequencing (CEL-seq) (Hashimshony et al., 2012) in 2012, Smart-seq2 (Picelli et al., 2013) in 2013, CytoSeq (Fan et al., 2015), Drop-seq (Klein et al., 2015), indexing Droplets (inDrop) (Macosko et al., 2015) in 2015, and 10x genomics (Hedlund and Deng, 2018) in 2017. The advantages of these methods have been disscused before (Picelli et al., 2014; Picelli, 2017), here we list part of them in Supplementary Table 1.

The widely used methods in nerve systems are Drop-seq, 10x genomics, and Smart-seq2. For instance, Drop-seq was used for identifying cortical inhibitory interneurons during development (Mayer et al., 2018), and 10x genomics was applied to explore the interneuron origin and molecular diversity in the human fetal brain (Yu et al., 2021). Through Smart-seq2, novel regeneration-associated genes that significantly promote axon regeneration were found in mouse retinal ganglion cells (Li et al., 2022). Among these methods, 10x genomics is most frequently applied for high throughput sequencing for its unique advantages in the identification of cell heterogeneity, especially the rare cell types. While Smart-seq2 allows *de novo* genome assembly and the sequencing of transcripts in their entirety, and thus provides detailed information on the particular cell type or specific regions (Li et al., 2016; Nichterwitz et al., 2016).

Here we summarize the application of single-cell technology in the PNS, especially neurons and glia in normal or injury states. This review helps to achieve a better understanding of cellular compositions and functions in the PNS.

## 2. Constitutions of the PNS in the naïve state

The sensory fibers of the neurons in PNS convey sensation from specific sense structures to the CNS and allow motor commands. Those neurons flock to sensory ganglia performing specific functions, such as the sensations of temperature, itchiness, and pain (detected by somatosensory neurons), as well as viscera physiology and gustation (detected by visceral sensory neurons). In the past, sensory neurons have been classified directly by their size (Wang et al., 1994), or response to stimuli (Ma, 2010). Until the advent of single-cell sequencing, we were able to classify neurons according to their molecular features. Following, we will discuss the applications of single-cell sequencing in neurons and glia in PNS.

### 2.1. Diversity of neurons

A recent study analyzed nearly half a million single cells from mouse nerve tissues and obtained a comprehensive survey of the mammalian nervous system (Zeisel et al., 2018). Cells in the nervous system are classified and assigned to seven major classes, i.e., neurons, oligodendrocytes, astrocytes, ependymal cells, peripheral glia (e.g., Schwann cells, satellite glia, and enteric glia), immune cells, and vascular cells (Zeisel et al., 2018). Peripheral neurons are split into sensory, sympathetic, and enteric subdivisions, which correspond to their functional and anatomical classification. Furthermore, sensory neurons are divided into three sub-types of neurofilament-containing neurons (NF, *Nefh*), six sub-types of non-peptidergic nociceptors (NP, *Mrgprd*), and eight sub-types of peptidergic nociceptors (PEP, *Ntrk1*). Sympathetic neurons are classified into two main branches, which are five sub-types of noradrenergic neurons (NOR, *Pthlh*) and two sub-types of cholinergic neurons (CHO, *Vip*). And a total of nine sub-types of enteric neurons (ENT1-3, *Nos1*; ENT1-9, *Chat*) are defined. The main sub-types of peripheral neurons are listed in Supplementary Table 2.

Somatosensory neurons in dorsal root ganglia (DRG) are thoroughly investigated due to the convenient collection of DRG tissues. As early as 2015, 799 single cells collected from the mouse lumbar DRG were sequenced with sequencing reads mapped to 3,574 ± 2,010 genes per cell (Usoskin et al., 2015). Cell populations are clustered into non-neuronal cells and 11 principal sensory neuron types, containing five clusters of NF, three clusters of NP, two clusters of PEP, and a cluster of tyrosine hydroxylase containing neurons (TH, *Th*) (Usoskin et al., 2015). Single-nucleus RNA sequencing of 141,093 DRG nuclei offers more persuasive data on neuron heterogeneity. In the naïve state, DRG neurons can be clustered into 9 sub-types: C-fiber LTMRs (cLTMR1, *Fam19a4^+^/Th^+^*), putative cLTMR2 (p_cLTMR2, *Fam19a4^+^/Th^–^*), PEP1, PEP2, NP, NF1, NF2, NF3, and *Sst*^+^ pruriceptors (SST, *Sst*^+^) (Renthal et al., 2020). Main neuronal types commonly recognized in both studies and the differences in classified sub-types in these studies may be caused by different cell separation methods, the depth of sequencing, and late-time analyses (van den Brink et al., 2017).

Cell clustering of somatosensory neurons during development demonstrates the dynamic changes of somatosensory neurons (Sharma et al., 2020). Shortly after DRG formation, single-cell sequencing reveals three principal cell types in embryonic day 11.5 (E11.5): neural crest progenitors (NCPs), sensory neuron progenitors (SNPs), and nascent, postmitotic sensory neurons (Avil^+^). Avil^+^ neurons exist in a transcriptionally unspecialized state, which serves as the starting point for sub-type diversification from E12.5 to adult. Somatosensory neurons in young postnatal (P) adults (P 28-40) can be classified into more groups, including five sub-types of LTMR, six sub-types of CGRP (*Calca*), Mrgprd^+^ (*Mrgprd*), proprioceptors (*Ntrk3*), somatostatin, and cold thermoceptors *(Trpv1)*. A consolidated view of the transcriptional maturation of principal somatosensory neuron sub-types has been obtained using single-cell k-nearest neighbor graphs.^1^

Another high-coverage single-cell sequencing with 10,950 ± 1,218 genes per cell provides more neuronal size- and function-based clustering (Li et al., 2016). Corresponding to the traditional classification of PEP (C1-C2), TH (C3), and NP (C4-C5), small diameter DRG neurons (cross-sectional area < 800 μm^2^) are grouped to C1-C6. Large diameter DRG neurons are groups to C7-C10, which correspond to the traditional NF classification (Wang et al., 2021a,b). Through *in vivo* whole-cell patch clamp recording of single neurons, small DRG neurons in C1, C2, C4, C5, and C6 generally act as mechanoheat nociceptors (MHNs) and are mainly sensitive to noxious mechanical stimulus and heat. Large DRG neurons in C7-C9 are mechanically sensitive neurons, mechanoreceptors, and mechanical nociceptors, respectively (Wang et al., 2021a).

The polymodal sensor of these clusters indicates that the functional heterogeneity of DRG neurons cannot simply be divided by neuron sizes or transcriptomes. It is interesting to determine whether a stimulus sensors-based classification can be explained by mechanosensitive (MS) ion channels in neurons. To answer this question, Zheng et al. (2019) have employed genetic labeling to purify eight major DRG neuron sub-types (NP, PEP, LTMRs, proprioceptors). The whole-cell patch-clamp recordings reveal the diversity of firing patterns and action potential (AP) waveforms. Deep sequencing of these neuron sub-types excavates gene expression patterns and reveals that Kv families contribute to the outward currents. From another perspective, Parpaite et al. (2021) have mapped the data of patch sequencing to transcriptional clusters identified by previous single-cell sequencing and determined the MS characteristics of different types of DRG neurons. NF corresponds to 97% rapidly adapting (RA) current. NP1 subclass corresponds to all four types of MS currents. NP2 neurons have almost no slowly adapting (SA) current but a high amount of RA currents (42%). PEP1 subclass has intermediately adapting (IA) currents (56%) but no or little SA and ultra-SA current. The PEP2 subclass has ultra-SA (39%), RA (34%), and SA currents (23%), but almost no IA current. TH neurons show both RA and ultra-SA currents. At last, they show that *Piezo2*, instead of *Tmem120a* and *Tmem150c*, mediates MS currents in DRG neurons. Although the numbers of sequenced neurons are limited, it offers enlightenment on bridging molecular characteristics to function heterogeneity through transcriptome sequencing.

A comparison between human DRG and mouse DRG from RNA sequencing reflects the highly conserved evolutionary genetic signature of DRG neurons (Ray et al., 2018). However, human DRG neurons have different cell sub-types (Nguyen et al., 2021). Typically, human somatosensory neurons can be divided into 15 sub-types. Some sub-types of DRG neurons in humans show high similarity with mouse DRG neurons, including proprioceptors (*Ntrk3*), c-peptidergic nociceptors (*Ntrk1*), and cold thermoceptors. Some sub-types, especially H4, H9, and H12, are human-specific. Recently, single-cell spatial transcriptomics has been applied in human DRG to identify transcriptomic signatures of nociceptors (Tavares-Ferreira et al., 2022). Obtained human DRG neurons are assigned to 12 clusters, including 1 proprioceptor, 2 Aβ-LTMRs, 1 Aβ nociceptor, 1 Aδ-LTMR, one Aβ high-threshold mechanoreceptor (HTMR), 1 C-LTMR, and 5 C-nociceptor sub-types. Different DRG neuron clusters were identified from human, non-human primates and organoids from human iPSC (Mazzara et al., 2020; Kupari et al., 2021). The detailed clusters were listed in Supplementary Table 2. Moreover, sex DRG subpopulation in human DRG have been identified, including one female pruritogen receptor-enriched nociceptors (*CALCA^high^*).

In addition to DRG neurons, trigeminal ganglia (TG) neurons are highly specialized sensory neurons. Through RNA sequencing of lumbar DRG and TG neurons from Advillin-GFP transgenic mice (Lopes et al., 2017) and rats (Kogelman et al., 2017), more than 99% of genes have similar expressions. Single-cell sequencing has been conducted to determine whether the sub-type of TG neurons is also similar to that of DRG. In 2017, about 7,000 cells from mouse TG were identified through Drop-seq. A total of 13 distinct sub-types are related to mechanoreceptors (*Ntrk2*), C-LTMR (*Th*), thermoceptors (*Trpm8*), pruriceptors (*P2y1*), and nociceptors (*Trpv1*) have been identified (Nguyen et al., 2017). Another 10x genomics sequencing of about 5,500 cells from TG reveals a similar cell clustering and identifies 12 distinct sub-types including Aβ/δ-LTMR, C-LTMR, CGRP-α/ε/η/γ/θ/ζ, MrgprA3^+^, somatostatin^+^ and cold thermoceptors (Sharma et al., 2020). Certain conserved sub-types of neurons are thus recognized in DRG and TG. Similar to DRG neurons, RNA sequencing data of human and mouse TG reveal similar molecular features between different species (Flegel et al., 2015).

### 2.2. Glia

Peripheral glial cells contain Schwann cells (SCs), satellite glial cells (SGCs), and enteric glial cells (EGCs) (Jessen, 2004). Both of them develop from neural crest cells (NCCs), which migrate ventrally and give rise to all glial subtypes at E11 (Jacob et al., 2014). At nascent DRG, NCCs give rise to glial precursors and further differentiate into SGCs and immature SCs (Kastriti and Adameyko, 2017). At last, the immature SCs develop into myelinating and non-myelinating SCs, forming myelin and Remak bundles, respectively (Jessen et al., 2015). On the other hand, NCCs invade the bowel and migrate through the mesenchyme. During migration, these enteric neural crest-derived cells (ENCDCs) proliferate and differentiate into enteric neurons and enteric glial cells (EGCs), which finally condense into ganglia to form a network throughout the bowel (Lake and Heuckeroth, 2013). Next, the heterogeneity of SCs, SGCs, and EGCs from single-cell RNA sequencing will be discussed, as listed in Supplementary Table 3.

#### 2.2.1. SCs

Schwann cells surround axons, and obtain a comprehensive capacity for support, nutrition, myelination, and pro-inflammation. Single-cell transcriptomics of mice PNS distinguishes myelinating and non-myelinating SCs (Wolbert et al., 2020). Combining with bulk RNA sequencing (bulk-seq), Smart-seq, and 10x genomic sequencing of sciatic nerves from P1, P5, P14, and P60 mice, SCs can be divided into six specific sub-types: proliferating SCs (prol.SC, *Mki67*), immature SCs (iSC, *Ngfr*), pro-myelinating SCs (pmSC, *Ncmap*), myelinating SCs (mSC, *Mpz*), transition SCs (tSC, *Ncam1*), mature non-myelinating SCs [nm(R)SC, *Ncam1*]. At the early maturation stage of SCs at P1, there are only prol.SC, iSC, and pmSC sub-types. iSC disappears at P5. tSC appears at P14 and switches to mSC and nm(R)SC at P60. Predicted developmental trajectory demonstrates that myelinating SCs come from prol.SC-iSC-pmSC axle while non-myelinating SCs come from prol.SC-iSC-tSC axle (Gerber et al., 2021). However, cells in this study are obtained from Fluorescence-activated cell sorting (FACS), which may have limitations in potential subsampling and cell extraction efficiency.

By using single-nucleus RNA sequencing, a method with non-performance of cell sensitivity and little influence on cell-dissociation procedures (van den Brink et al., 2017; Nguyen et al., 2019), a recent study has revealed multiple sub-types of SCs in mouse sciatic nerves (Yim et al., 2022). Among identified SC clusters, cluster 4 expressing *Scn7a* and *Csmd1* and cluster five expressing *Marcks* and *Prnp* belong to nmSCs. Cluster 0 expresses high levels of *Plp1*, cluster 1 expresses *Lmna* and *Egr2*, cluster 2 expresses *Col23a1*, and cluster 3 co-expressing *Cldn14*, *Adamtsl1*, and *Pmp2* are mSCs. Cluster 3 SCs preferentially ensheath motor axons. Correspondingly, the numbers of the Pmp2^+^ SCs are reduced in amyotrophic lateral sclerosis (ALS) SOD1G93A mouse model as well as human ALS nerve samples. Another research of sciatic nerves and DRG tissues of neonatal rats separates SCs into four sub-types with diverse biological activities (Zhang et al., 2021b). *LOC100134871* and *Hbb* expressing sub-type 1 SCs have features of connective tissue cells. *Cldn19* and *Emid1* expressing sub-tpye 2 SCs have features of pmSC. *Timp3* and *Col5a3* expressing sub-type 3 SCs exist in a more dedifferentiated state similar to iSC. *Cenpf* and *Mki67* expressing sub-type 4 SCs are similar to the prol.SC (Zhang et al., 2021a,b). In addition, the percentage of these four sub-types of SC in the sciatic nerve and DRG is quite different. The high abundance of sub-type 3 SCs in sciatic nerves and low abundance of sub-type SCs in DRG indicate the different degrees of development of SCs at different anatomical localization.

#### 2.2.2. SGCs

Different from SCs which mainly surround axons, SGCs envelop the neuronal soma and account for the main population in the ganglia. However, the difficulty of separating SGCs from neurons or other cells caused SGCs to receive only modest scientific attention until the appearance of single-cell sequencing. Recently, 10x genomics sequencing of living cells from mouse DRG through FACS has identified various cell types in DRG, with SGCs accounting for about 30%. The specific marker genes for SGCs are *Kcnj10*, *Cdh19*, and *Fabp7* (Tasdemir-Yilmaz et al., 2021). There are two different sub-types of SGCs in DRG at P14, immature SGC and mature SGC. Both of them come from the glial precursors (GPs) at E14.

Four sub-types (clusters 1-4) of SGCs in adult DRG that are enriched in the different signal pathways and substance metabolisms have been identified (Avraham et al., 2021). Previous studies demonstrate that SGCs display a strongest transcriptional correlation with astrocytes rather than SCs, although both SGCs and SCs originate from GPs (Tasdemir-Yilmaz et al., 2021). When comparing each SGC sub-cluster with other glial cells, it is demonstrated that clusters 1 and 2 are unique SGC sub-types. *Aldh1l1* expressing cluster 3 SGCs share many common genes with astrocytes while *Scn7a* expressing cluster 4 SGCs are similar to myelinated SCs. Notably, SGCs in DRG represent distinct characteristics from in spiral ganglia (SG). SGCs in SG have high expressions of myelination-associated genes such as *Mpz*, *Mbp*, *Pmp22*, and *Egr2*. A possible reason is that the cell bodies of neurons in SG are highly myelinated (Hibino et al., 1999; Tasdemir-Yilmaz et al., 2021).

Similar to neurons, SGCs in mice, rats, and humans have similar transcriptional profiles (Avraham et al., 2022), but human SGC expresses a greater variety of ion channels and receptors compared to rodent SGC. The conserved functional properties between rodent and human demonstrate that rodents are suitable models for the investigation of human neurological disorders.

#### 2.2.3. EGCs

Enteric glial cells in the enteric nervous system (ENS) are the largest collection of glia outside of the CNS. EGCs are non-myelinating glial cells that have high similarity with astroglia and strong crosstalk with enteric neurons (Grubisic and Gulbransen, 2017). The first single-cell sequencing of the ENS at E12.5 identified three groups of cells, that is glial cells, neuronal cells, and cells that have both glial and neuron characters. These glial cells express normal glial markers like *Sox10*, *Erbb3*, and *Plp1* (Lasrado et al., 2017). Using *Wnt1Cre;R26Tomato* transgenic mice, two studies have identified different sub-types of enteric glia. Zeisel et al. (2018) have revealed the existence of seven distinct sub-types, including one sub-type of actively proliferating glia. Wright et al. (2021) have displayed 4 sub-types of enteric glia from adult mouse distal colon. In human embryos, six clusters of enteric glial cells have been identified (Drokhlyansky et al., 2020). Regretfully, there are no in-depth analyses of EGCs.

Based on the scRNA-seq data of the murine intestinal mesenchyme from Roulis et al. (2020), Baghdadi et al. (2022) have identified three subpopulations of EGCs, designated as EGC#0 (*Gfap^High^*), EGC#1 (*Plp1^High^*), and EGC#2. The *Gfap*^+^ sub-type EGCs are essential for intestinal stem cell activities and intestinal regeneration after injury. In human colonic stroma, four sub-types of EGCs (EGC#0-3) have been identified (Kinchen et al., 2018). Similarly, human EGC#0 and EGC#1 represent high *Gfap* and *Plp1*, respectively, indicating that the subpopulations of EGCs are also conserved between human and mouse. Moreover, it has been discovered that EGCs from humans with inflammatory bowel disease display high expression of *Gfap*, suggesting that *Gfap*^+^ EGCs reveal a great influence on intestinal homeostasis and regeneration.

## 3. Changes of the PNS following peripheral nerve injury

Nerve injury induces motor, sensory, and autonomic dysfunctions, largely impairing patients’ quality of life and causing a great socioeconomic burden (Gu et al., 2011). A deeper understanding of cellular alternations after nerve injury may facilitate its clinical treatment. The PNS, compared to the CNS, obtains certain intrinsic growth capacities following nerve injury (He and Jin, 2016). Therefore, changes after peripheral nerve injury have been well-deciphered from the single-cell aspect, aiming to decode underlying cellular mechanisms underlying nerve regeneration.

Smart-seq2 single-cell sequencing of DRG neurons at 3 and 7 days after sciatic nerve transection demonstrates that small unmyelinated neurons non-peptidergic nociceptors, medium-sized lightly-myelinated neurons peptidergic nociceptors, and large diameter myelinated neurons have heterogeneous responses to axonal injury (Hu et al., 2016). Numerous injury-responsive genes, including classic regeneration-associated genes *Atf3* and *c-Jun* as well as novel regeneration-associated genes, are identified in these three sub-types of neurons (Nguyen et al., 2019). Many differentially expressed genes in non-peptidergic nociceptors are found to be highly related to cell survival and death (Nguyen et al., 2019). Elevated abundances of neural damage marker gene *Atf3* and other injury-associated genes such as *Gal* and *Sox11* are also detected in trigeminal neurons after partial infraorbital transection of the trigeminal nerve spared nerve injury, indicating the repaid induction of a shared injured-neuron transcriptional state (Nguyen et al., 2019). These injured trigeminal neurons (Supplementary Table 2, red labeled) exist in an injury-related state for a long time and then slowly reacquire normal genetic profiles (Nguyen et al., 2019).

**FIGURE 1:**
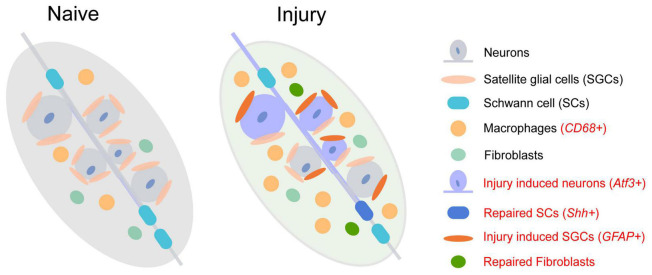
The change of cell subtypes in dorsal root ganglia following sciatic nerve injury. The injury-induced cell subtype is labeled in red.

Single-nucleus RNA sequencing of sensory ganglia after peripheral nerve crush or transection defined an injury state of DRG neurons (Renthal et al., 2020). Nine sub-types of neurons defined in the naïve state present a similar response to injury and a common up-regulation of *Atf3*. *Atf3* drives transcriptional reprogramming and regeneration of DRG neurons after injury. A similar injury state of DRG neurons was also identified in another 10x Genomics scRNA-seq of L4-5 DRG (Wang et al., 2021a). Novel clusters of neurons (*Atf3*/*Gfra3*/*Gal* neurons, *Atf3*/*Mrgprd* neurons, and *Atf3*/*S100b*/*Gal* neurons) are specifically induced by nerve injury (Figure 1 and Supplementary Table 2, red labeled).

Although the transcriptional responses in non-neuronal DRG cell types are quite different from neuronal sub-types, new clusters of repair Schwann cells (Figure 1 and Supplementary Table 3, red labeled) and fibroblasts are observed after nerve injury (Renthal et al., 2020). The repair Schwann cells are specifically labeled by *Shh*, showing an increased amount from 2 days after spinal nerve transection but not after sciatic nerve transection or crush. The increase of macrophages following peripheral nerve injury is also detected as a significant infiltration of *Lyz2*^+^ immune cells into DRG 7 days after injury (Jager et al., 2020; Renthal et al., 2020). Epineurial *Relmα*^+^*Mgl1*^+^ snMacs do not extensively react to nerve injury while endoneurial *Relmα*^–^*Mgl1*^–^ snMacs produce monocyte chemoattractants and recruit monocyte-derived macrophages to the injured site (Ydens et al., 2020). Among the four distinct sub-types of mesenchymal cells in normal and injured peripheral nerves, endoneurial *Pdgfra*^+^ nerve mesenchymal cells, a cellular population increased after nerve injury, obtain precursor properties and facilitate tissue repair (Carr et al., 2019). These *Pdgfra*^+^ nerve mesenchymal cells from injured rodent nerves secret paracrine factors, such as *Angpt1*, *Ccl11*, and *Vegfc*, and promote axon growth (Toma et al., 2020).

Surprisingly, as the most abundant glia in DRG, the number of SGCs shows little change after injury. Following nerve injury, upregulated genes involved in lipid metabolism and the immune system in SGCs contribute to promoting axon regeneration of DRG neurons (Avraham et al., 2020). However, the numbers and components of sub-types of SGCs are diverse in DRG after sciatic nerve crush (SNC), dorsal root crush (DRC), and spinal cord injury (SCI) (Avraham et al., 2021). Two new sub-types appeared after injury (Supplementary Table 3, red labeled), in which a large cluster 6 emerged after SNC, and cluster 7 emerged after SCI. Through transcription factor binding site analysis of differentially expressed genes, it is demonstrated that cluster 6 adopts a more plastic state after SNC, which might contribute to axon regeneration.

## 4. Conclusion and future perspectives

It is obvious from the above description that some tissues exhibit different cell types or different makers of specific cell types, which may due to different sequencing technology or analysis methods. In order to reduce this variation, a combination of bulk-seq, high throughput sequencing, and deep sequencing can provide more convincing data (Gerber et al., 2021). In addition, the comprehensiveness of the database and unbiased method of analysis can also help us to better compare the data from different studies.

Practically, the emergence of single-cell multi-omics allows simultaneous transcriptome, genome, epigenome, and proteome profiling and thus provides a path to decipher the underlying regulatory mechanism (Hu et al., 2018). Sun et al. (2021) profile the epigenome (H3K27me3 and H3K4me3) and transcriptome in mouse embryonic stem cells jointly, revealing epigenomic and transcriptomic of the same single cell and thus disclose gene regulatory mechanistic that influence cell fate. BRAIN Initiative Cell Census Network utilizes single-cell transcriptomes, chromatin accessibility, DNA methylomes, spatially resolved single-cell transcriptomes, morphological and electrophysiological properties, and cellular resolution input-output mapping and achieves a comprehensive understanding of mammalian primary motor cortex (BRAIN Initiative Cell Census Network (BICCN), 2021). However, single-cell multi-omics mainly focus on the CNS, and their application in the PNS is relatively limited. The methylation and metabonomics of DRG have been sequenced at the bulk level (Weng et al., 2018; Zhang et al., 2022). Mapping these dates to single-cell transcriptomes may offer enlightenment to discover specific responded cell sub-types under naïve or disease states.

To conclude, we hope that more numerous and effective technologies, databases, and analysis methods can emerge to uncover and describe the development and diseases of the nervous system. At the same time, we also hope that existing technologies can be more applied in PNS, to provide data and theoretical support for identifying potential targets in the treatment of nerve injuries.

## Author contributions

LZ and WH wrote the manuscript. SY revised and modified. All authors read and approved the final manuscript.

## Footnotes

1
https://kleintools.hms.harvard.edu/tools/springViewer_1_6_dev.html?datasets/Sharma2019/all
