# Supplementary material for: Cellular complexity of the peripheral nervous system: Insights from single-cell resolution
Source: Front Neurosci. 2023 Mar 14;17:1098612. doi: 10.3389/fnins.2023.1098612 (PMC10043217; doi:10.3389/fnins.2023.1098612)
Supplement: Supplementary file 1 [file Table_1.docx]

Table 1. The advantages of different single-cell sequencing methods.

| Methods | Year | Advantages | References |
| --- | --- | --- | --- |
| STRT-seq | 2011 | Count the number of unique transcripts expressed in each cell and tell them apart from PCR duplicates | (Picelli, 2017) |
| Smart-seq | 2012 | Provide even read coverage  Enhance detailed analyses of alternative transcript isoforms and identification of single-nucleotide polymorphisms | (Ramskold et al., 2012) |
| Smart-seq 2 | 2013 | Increase cDNA yield  Enhance sensitivity  Reduce technical biases and variability | (Picelli et al., 2014) |
| CEL-seq | 2012 | Decrease reading preference | (Picelli, 2017) |
| CytoSeq | 2015 | Capture flexibility | (Picelli, 2017) |
| Drop-seq | 2015 | Capture efficiently  Cost low | (Picelli, 2017) |
| inDrop | 2015 | Sequence large numbers of cells from heterogeneous populations in an extremely fast way | (Picelli, 2017) |
| 10x genomics | 2017 | High throughput  Cover a large number of cells to easily detect rare cell types | (Wang et al., 2021c) |
