# Supplementary material for: Cellular complexity of the peripheral nervous system: Insights from single-cell resolution
Source: Front Neurosci. 2023 Mar 14;17:1098612. doi: 10.3389/fnins.2023.1098612 (PMC10043217; doi:10.3389/fnins.2023.1098612)
Supplement: Supplementary file 2 [file Table_2.docx]

Table 2. The sub-type of neurons in DRG and TG.

| Sample source | Methods | Number of sequenced cells/nuclei | Main findings | Reference |
| --- | --- | --- | --- | --- |
| Mouse whole nerve system | 10x genomics | 509,876 | NF1-3  NP1-6  PEP1-8  NOR1-5  CHO1-2  ENT1-9 | (Zeisel et al., 2018) |
| Mouse lumbar DRG | Smart-seq2 | 799 | NF1-5  NP1-3  PEP1-2  TH | (Usoskin et al., 2015a) |
| Mouse lumbar DRG | single-nucleus RNA sequencing | 141093 | cLTMR1  p_cLTMR2  PEP1-2  NF1-3  NP  SST  Injury-induced state (injury state) | (Renthal et al., 2020) |
| Mouse DRG | 10x genomics | More than 120, 000 | E11.5:  NCPs, SNPs, nascent Avil^+^ sensory neurons  E12.5-adult:  LTMRs  CGRP^+^ neurons  Mrgprd^+^ polymodal nociceptors  proprioceptors  Sst^+^ pruriceptors  cold thermoreceptors | (Sharma et al., 2020) |
| Mouse lumbar 4-5 DRG | Smart-seq2 & 10x genomics | 203 & 36,810 | C1-C10  SNIIC1–3 (injury state) | (Wang et al., 2021a) |
| Human  DRG | Spatial transcriptomics | 37,208 barcodes | proprioceptor  Aβ-LTMR  Aβ nociceptor  Aδ-LTMR  Aβ-HTMR  C-LTMR  C-nociceptor sub-types | (Tavares-Ferreira et al., 2022) |
| Mouse TG | 10x genomics | 5556 | LTMRs  CGRP^+^ neurons  Mrgprd^+^ polymodal nociceptors  Sst^+^ pruriceptors  cold thermoreceptors | (Sharma et al., 2020) |
| Mouse TG | Drop-seq | 7000 | C1-13 | (Nguyen et al., 2017) |
| Mouse TG | Drop-seq & 10x genomics | more than 7500 | C1-13  I1-2 (injury state) | (Nguyen et al., 2019) |
| Human L4-5 DRG | 10x genomics | 1837 | H1-H15 | (Nguyen et al., 2021a) |
| DRG organoids from human induced pluripotent stem cells | 10x genomics | 5363 | C1: neuronal progenitors  C2 and C3: Proprioceptors (putative)  C4: Nociceptors  C5-C8: Mechanoreceptors (putative) | (Mazzara et al., 2020) |
| DRG from adult Rhesus macaques | STRT-2i-seq | 4742 | NP1-3  PEP1-3  A-LTMRs  C-LTMRs  TrpM8^high^ | (Kupari et al., 2021) |
