# Supplementary material for: Cellular complexity of the peripheral nervous system: Insights from single-cell resolution
Source: Front Neurosci. 2023 Mar 14;17:1098612. doi: 10.3389/fnins.2023.1098612 (PMC10043217; doi:10.3389/fnins.2023.1098612)
Supplement: Supplementary file 3 [file Table_3.docx]

Table 3. The sub-type of glial cells in the PNS.

| Cell type | Sample source | | Methods | | Number of sequenced cells/nuclei | Main findings | Reference |
| --- | --- | --- | --- | --- | --- | --- | --- |
| SC | Mouse sciatic nerves | Smart-seq2 & 10x genomics | | 384 & 23767 | | prol. SC  nm(R)SC  iSC  tSC  pmSCs  mSC | (Gerber et al., 2021) |
|  | Mouse sciatic nerves | 10x genomics | | 20,658 | | cluster 1  cluster 2  cluster 3  cluster 4  cluster 5  cluster 6 | (Yim et al., 2022) |
|  | Rat sciatic nerves & DRG | 10x genomics | | 19,202 | | sub-type 1-4 | (Zhang et al., 2021b) |

|  | Mouse lumbar DRG | 10x genomics | 141,093 | Schwann_M  Schwann_N  Repair Schwann (injury state) | (Renthal et al., 2020) |
| --- | --- | --- | --- | --- | --- |
| SGC | Mouse whole nerve system | 10x genomics | 509,876 | SATG1 (proliferating)  SATG2 | (Zeisel et al., 2018) |
|  | Mouse cochlea & lumbar DRG & sciatic nerve | inDrop | 52,323 | g1-11 | (Tasdemir-Yilmaz et al., 2021) |
|  | Mouse DRG | 10x genomics | 25,154 | cluster 1  cluster 2  cluster 3  cluster 4  cluster 5  cluster 6 (injury state)  cluster 7 (injury state) | (Avraham et al., 2021) |
| EGC | Mouse whole nerve system | 10x genomics | 509,876 | ENT G1-G7 | (Zeisel et al., 2018) |
|  | Mouse distal colon | 10x genomics | 1520 | glia 1-4 | (Wright et al., 2021) |
|  | Mouse colon | RAISIN RNA-Seq with SMART-Seq2 | 5696 | glia 1-3 | (Drokhlyansky et al., 2020) |
|  | Mouse intestinal mesenchyme | Drop-seq | 3179 | EGC#0-2 | (Baghdadi et al., 2022; Roulis et al., 2020) |
|  | Human colon | MIRACL-Seq | 6054 | glia 1-6 | (Drokhlyansky et al., 2020) |
